# Investigation of spleen CXCR4 expression by [^68^Ga]Pentixafor PET in a cohort of 145 solid cancer patients

**DOI:** 10.1186/s13550-021-00822-6

**Published:** 2021-08-21

**Authors:** Richard Lewis, Stefan Habringer, Malte Kircher, Maike Hefter, Caroline Anna Peuker, Rudolf Werner, Valëza Ademaj-Kospiri, Alexander Gäble, Wolfgang Weber, Hans-Jürgen Wester, Andreas Buck, Peter Herhaus, Constantin Lapa, Ulrich Keller

**Affiliations:** 1 Department of Hematology, Oncology and Cancer Immunology, Campus Benjamin Franklin, Charité - Universitätsmedizin Berlin, Corporate Member of Freie Universität Berlin and Humboldt-Universität zu Berlin, Hindenburgdamm 30, 12203 Berlin, Germany; 2 Berlin Institute of Health (BIH), Berlin, Germany; 3 Nuclear Medicine, Medical Faculty, University of Augsburg, Augsburg, Germany; 4 Clinic and Policlinic for Internal Medicine III, School of Medicine, Technical University of Munich, Munich, Germany; 5 Department of Nuclear Medicine, University of Würzburg, Würzburg, Germany; 6 Clinic for Nuclear Medicine, School of Medicine, Technical University of Munich, Munich, Germany; 7 Chair of Pharmaceutical Chemistry, Technical University of Munich, Garching, Germany; 8 German Cancer Consortium (DKTK), Partner Site Berlin; and German Cancer Research Center (DKFZ), Heidelberg, Germany; 9 Max-Delbrück-Center for Molecular Medicine, Berlin, Germany

**Keywords:** Solid tumors, Clinical studies, Retrospective studies, Molecular imaging, PET, CXCR4, Pentixafor, Spleen, Uptake

## Abstract

**Background:**

The chemokine receptor CXCR4 is frequently overexpressed and associated with adverse prognosis in most hematopoietic malignancies and solid cancers. Recently, CXCR4 molecular imaging using the CXCR4-specific positron emission tomography (PET) tracer Pentixafor ([^68^Ga]Pentixafor) has become a well-established method to non-invasively measure CXCR4 expression in vivo. In previous Pentixafor imaging studies, highly variable CXCR4 tracer uptake to the spleen was observed.

**Results:**

We investigated the hypothesis that enhanced spleen [^68^Ga]Pentixafor uptake and thus CXCR4 expression in patients with solid tumors would indicate an activated spleen state and/or an association with clinical and prognostic features and survival parameters. In this retrospective study, [^68^Ga]Pentixafor-PET images and patient records of 145 solid tumor patients representing 27 cancer entities were investigated for an association of spleen [^68^Ga]Pentixafor uptake and clinical characteristics and outcome. Based on this assessment, we did not observe differences in clinical outcomes, measured by progression-free survival, overall survival and remission status neither within the entire cohort nor within subgroups of adrenal cancer, desmoplastic small round cell tumor, neuroendocrine tumors, non-small cell lung cancer, small cell lung cancer and pancreatic adenocarcinoma patients. No tumor entity showed especially high levels of spleen [^68^Ga]Pentixafor uptake compared to others or a control cohort. However, when investigating laboratory parameters, there was a positive correlation of high spleen [^68^Ga]Pentixafor uptake with leukocyte and/or platelet counts in neuroendocrine tumors, non-small cell lung cancer and small cell lung cancer.

**Conclusion:**

Spleen [^68^Ga]Pentixafor uptake was not associated with stage of disease and clinical outcomes in solid tumor patients. We identified positively associated platelet and/or leukocyte counts with spleen [^68^Ga]Pentixafor uptake in neuroendocrine tumors, non-small cell lung cancer and small cell lung cancer, suggesting that splenic CXCR4 expression could possibly play a role in systemic immunity/inflammation in some types of solid tumors or a subgroup of patients within solid tumor entities.

**Supplementary Information:**

The online version contains supplementary material available at 10.1186/s13550-021-00822-6.

## Background

C-X-C motif chemokine receptor type 4 (CXCR4) and its main ligand stromal cell-derived factor 1 (SDF-1), have pivotal roles in hematopoiesis, cardiovascular development and homing of immune cells [1–3]. Thus, physiologically CXCR4 is highly expressed on hematopoietic cells such as B and T cells [2, 4]. In malignancies, CXCR4 has been shown to be overexpressed as compared to non-malignant control tissue and, moreover, to be associated with adverse prognosis in many different types of cancer [5–9], suggesting a role of CXCR4 as a tumor driver. Additionally, there is a plethora of data elucidating the critical function and likely complex role of CXCR4 for microenvironment, metastasis and dissemination of tumors [10–13]. [^68^Ga]Pentixafor positron emission tomography (PET) is a well-established method to image CXCR4 expression of hematopoietic and solid tumors in vivo [14–16] and previous studies with [^68^Ga]Pentixafor have revealed a wide heterogeneity of tumor CXCR4 expression within different entities of cancer [17–19]. Furthermore, in some entities, patients with high levels of tumor [^68^Ga]Pentixafor uptake exhibited unfavorable outcomes [20]. During previous studies in solid tumor patients using [^68^Ga]Pentixafor, we observed a wide variance not only of tumor, but also spleen CXCR4 expression [21]. In recent years, evidence of the importance of not only the local tumor microenvironment, but also the impact of systemic immunity on the suppression of anti-tumor immune responses and hence tumor progression has accumulated, shedding light on more complex interactions between tumor cells and primary as well as secondary lymphoid organs [22–24]. Various solid tumors for instance induce tumor-associated extra-medullary hematopoiesis in the spleen, associated with adverse patient outcomes [25, 26]. Furthermore, suppression of anti-tumor immunity is, along with other complex mechanisms, majorly regulated through immunosuppressive myeloid-derived suppressor cells (MDSCs), which originate in the spleen [27–29]. Tumor infiltrating MDSCs are known to express high levels of CXCR4 and to migrate towards a SDF-1 gradient [30, 31]. Splenic MDSCs are known to express CXCR4 as well, but the role of CXCR4 in the spleen for MDSC differentiation and release to peripheral sites remains unclear. Taken together, there is strong evidence that underlines the pivotal roles of the spleen and CXCR4 in the immune response to tumors. Hence, we hypothesized one explanation for the variability of spleen CXCR4 expression in solid tumor patients could potentially be an activated state of spleen, through tumor-related effects, associated with suppression of anti-tumor immunity and potentially adverse clinical outcomes. To this point, no large study investigating CXCR4 enrichment in spleens of solid tumor patients has been conducted and the relevance of these differences for clinical outcomes remains unclear. Thus, we aimed to investigate and report a large retrospective analysis evaluating clinical outcomes and laboratory parameters in relation to spleen CXCR4 expression of 145 patients with solid tumors and 5 control patients, which had previously been imaged with the CXCR4-specific PET tracer [^68^Ga]Pentixafor.

## Results

### Patient and control cohorts and their primary characteristics

We retrospectively included 145 solid tumor patients in this study, which had previously undergone imaging with [^68^Ga]Pentixafor-PET/computed tomography (CT). Furthermore, we included 5 control patients, of whom 3 were diagnosed with Conn’s adenoma and 2 were diagnosed as healthy. The group of tumor patients comprised 27 different entities and the largest groups of patients included 31 adrenal cancers, 25 neuroendocrine tumors, 13 small cell lung cancers (SCLC), 9 desmoplastic small round cell tumors (DSRCT), 9 pancreatic adenocarcinomas and 9 non-small cell lung cancers (NSCLC). In this cohort, 89 (61.4%) patients were male and 56 (38.6%) female and the mean age was 58.8 ± 14.7 years. At the time of [^68^Ga]Pentixafor-PET imaging 28 (19.3%) patients had limited stage of disease (T1/T2 without distant metastasis) and 62 (42.8%) patients advanced disease stage (T3/T4 or distant metastases). In 55 (37.9%) patients, the exact stage of disease at the time of imaging could not be determined retrospectively (Table 1).

**Table 1:** Primary characteristics of patients and controls

Entity	*n*	Male (*n*/%)	Female (*n*/%)	Age (mean ± SD)	Stage limited/advanced/unknown (*n*/%)
All tumors	145	89 (61.4%)	56 (38.6%)	58.8 ± 14.70	28 (19.3%)/62 (42.8%)/55 (37.9%)
Adrenal cancers	31	13 (41.9%)	18 (58.1%)	51.3 ± 9.97	5 (16.1%)/19 (61.3%)/7 (22.6%)
Atypical carcinoid of lung	1	0 (0.0%)	1 (100.0%)	58.0 ± 0.00	0 (0.0%)/0 (0.0%)/1 (100.0%)
Breast cancer (non-neuroendocrine)	2	0 (0.0%)	2 (100.0%)	59.5 ± 1.50	0 (0.0%)/0 (0.0%)/2 (100.0%)
Cholangiocarcinoma	4	3 (75.0%)	1 (25.0%)	70.3 ± 5.17	1 (25.0%)/2 (50.0%)/1 (25.0%)
Colorectal cancer	1	1 (100.0%)	0 (0.0%)	60.0 ± 0.00	1 (100.0%)/0 (0.0%)/0 (0.0%)
CUP (non-neuroendocrine)	3	1 (33.3%)	2 (66.7%)	74.3 ± 8.06	0 (0.0%)/2 (66.7%)/1 (33.3%)
DSRCT	9	9 (100.0%)	0 (0.0%)	29.4 ± 8.90	0 (0.0%)/0 (0.0%)/9 (100.0%)
Ewing’s sarcoma	1	1 (100.0%)	0 (0.0%)	53.0 ± 0.00	0 (0.0%)/1 (100.0%)/0 (0.0%)
Granulosa cell tumor	1	0 (0.0%)	1 (100.0%)	72.0 ± 0.00	1 (100.0%)/0 (0.0%)/0 (0.0%)
Hepatocellular carcinoma	6	3 (50.0%)	3 (50.0%)	61.3 ± 7.16	3 (50.0%)/1 (16.7%)/2 (33.3%)
Laryngeal cancer	1	1 (100.0%)	0 (0.0%)	72.0 ± 0.00	0 (0.0%)/0 (0.0%)/1 (100.0%)
Malignant paraganglioma	1	1 (100.0%)	0 (0.0%)	49.0 ± 0.00	0 (0.0%)/1 (100.0%)/0 (0.0%)
MANEC	2	1 (50.0%)	1 (50.0%)	65.5 ± 2.50	0 (0.0%)/2 (100.0%)/0 (0.0%)
Melanoma	2	2 (100.0%)	0 (0.0%)	66.0 ± 4.00	0 (0.0%)/0 (0.0%)/2 (100.0%)
Mesothelioma	6	5 (83.3%)	1 (16.7%)	69.3 ± 9.59	1 (16.7%)/2 (33.3%)/3 (50.0%)
Neuroendocrine tumors	25	17 (68.0%)	8 (32.0%)	66.3 ± 10.57	3 (12.0%)/14 (56.0%)/8 (32.0%)
NSCLC	9	8 (88.9%)	1 (11.1%)	59.3 ± 8.56	4 (44.4%)/5 (55.6%)/0 (0.0%)
Osteosarcoma	1	1 (100.0%)	0 (0.0%)	32.0 ± 0.00	1 (100.0%)/0 (0.0%)/0 (0.0%)
Ovarial cancer	1	0 (0.0%)	1 (100.0%)	73.0 ± 0.00	1 (100.0%)/0 (0.0%)/0 (0.0%)
Pancreatic adenocarcinoma	9	6 (66.7%)	3 (33.3%)	65.3 ± 12.89	2 (22.2%)/1 (11.1%)/6 (66.7%)
Prostate cancer	2	2 (100.0%)	0 (0.0%)	64.0 ± 8.00	0 (0.0%)/1 (50.0%)/1 (50.0%)
Renal cell carcinoma	4	3 (75.0%)	1 (25.0%)	71.8 ± 12.75	1 (25.0%)/2 (50.0%)/1 (25.0%)
Sarcoma	7	3 (42.9%)	4 (57.1%)	45.7 ± 18.90	2 (28.6%)/1 (14.3%)/4 (57.1%)
Salivary gland cancer	1	1 (100.0%)	0 (0.0%)	52.0 ± 0.00	0 (0.0%)/0 (0.0%)/1 (100.0%)
SCLC	13	6 (46.2%)	7 (53.8%)	62.2 ± 11.41	2 (15.4%)/7 (53.8%)/4 (30.8%)
Thyroid cancer	1	1 (100.0%)	0 (0.0%)	61.0 ± 0.00	0 (0.0%)/1 (100.0%)/0 (0.0%)
Undifferentiated cancer of thymus	1	0 (0.0%)	1 (100.0%)	41.0 ± 0.00	0 (0.0%)/0 (0.0%)/1 (100.0%)
Controls					
Conn’s adenom	3	3 (100.0%)	0 (0.0%)	57.7 ± 7.93	n.a
Healthy controls	2	1 (50.0%)	1 (50.0%)	65.5 ± 2.50	n.a

Table indicates the number of patients and control patients (*n*) separated by type of tumor entity, indicating the respective number and frequency of male and female patients (*n*/%), mean age of patients in years (mean ± SD) as well as the number and frequency of patients with limited, advanced and unknown tumor stage (*n*/%)

### Spleen [^68^Ga]Pentixafor uptake levels show a great variance and are not associated with prior systemic treatment

**Fig. 1:**
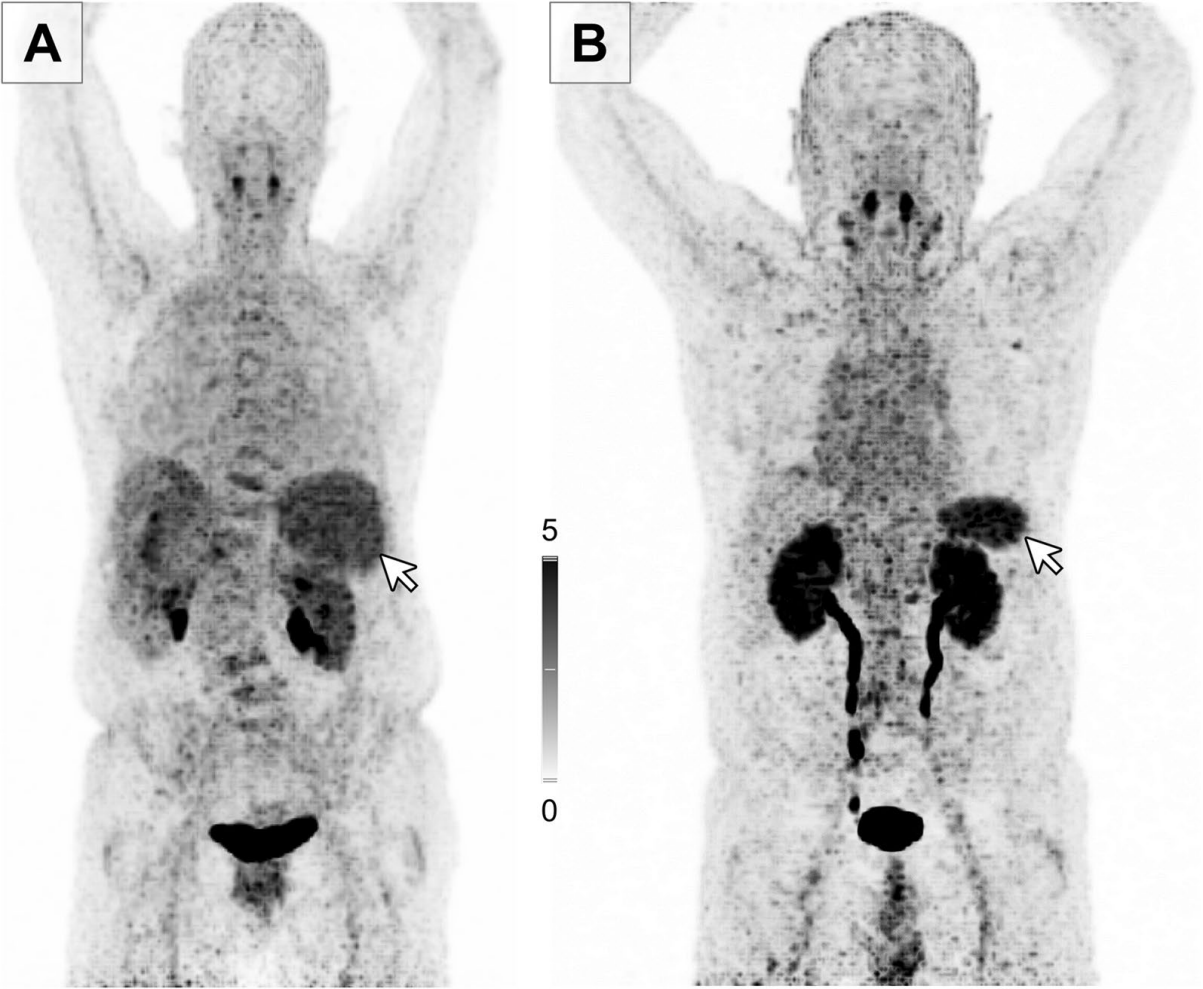
Patients with solid tumors present with different levels of [^68^Ga]Pentixafor uptake in spleen. **A** Representative image of a 68-year-old female patient presenting with hepatocellular carcinoma and low spleen CXCR4 expression as measured by [^68^Ga]Pentixafor uptake. **B** Representative image of a 68-year-old male patient presenting with a neuroendocrine tumor and high spleen CXCR4 expression as measured by [^68^Ga]Pentixafor uptake

We determined the maximum standardized uptake value (SUV) for [^68^Ga]Pentixafor of patients’ spleens (SUV_max_Spleen). Patients showed a great variance of splenic [^68^Ga]Pentixafor uptake with a mean SUV_max_Spleen of 8.50 ± 4.67 (range, 2.00–56.00). To rule out differences in uptake, we decided to normalize spleen uptake, by dividing the maximum splenic [^68^Ga]Pentixafor uptake by the mean hepatic uptake, therefore using the liver as a reference organ (SUV_max_Spleen/SUV_mean_Liver; spleen-to-liver ratio, SLR) and used this parameter for further analysis of spleen CXCR4 expression. The mean SLR was 6.19 ± 2.28 (range, 0.77–16.44). Examples of patients with low and high SLR are given in Fig. 1.

**Fig. 2:**
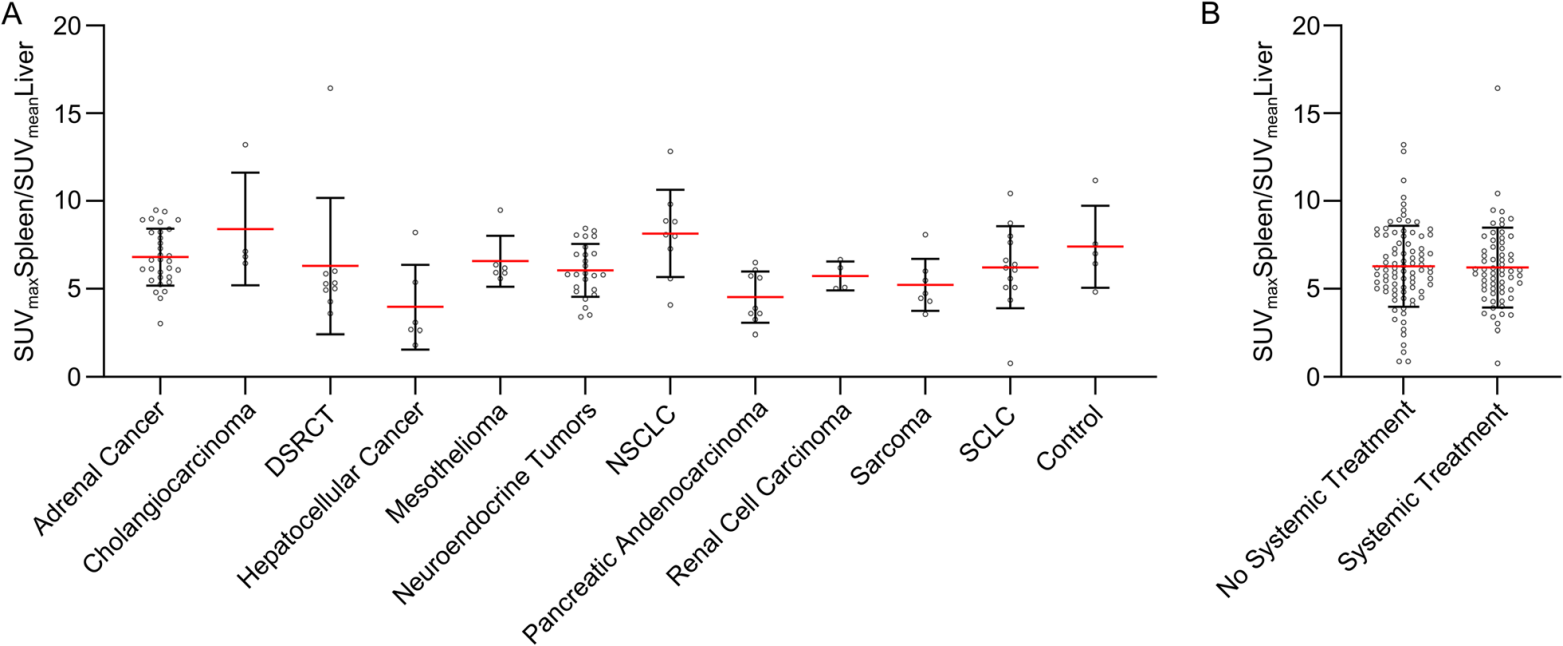
Spleen [^68^Ga]Pentixafor uptake levels show a wide variety within different entities, and are not affected by systemic treatment. **A** Comparison of spleen-to-liver ratios (SUV_max_Spleen/SUV_mean_Liver) of patients with adrenal cancer, *n* = 31; cholangiocarcinoma, *n* = 4; DSRCT, *n* = 9; hepatocellular carcinoma, *n* = 6; mesothelioma, *n* = 6; neuroendocrine Tumors, *n* = 25; NSCLC, *n* = 9; pancreatic adenocarcinoma, *n* = 9; renal cell carcinoma, *n* = 4; sarcoma, *n* = 7; SCLC, *n* = 13 and control, *n* = 5. **B** Comparison of spleen-to-liver ratios (SUV_max_Spleen/SUV_mean_Liver) of patients of all tumor entities included in the study, comparing patients without ongoing (no systemic treatment, *n* = 85) to those with an ongoing systemic therapy or systemic treatment within the last month prior to imaging (systemic treatment, *n* = 63)

As we observed solid cancer patients with very high and such with low spleen [^68^Ga]Pentixafor uptake expression, we sought to address the question, whether some solid tumor entities were associated with notably higher levels of chemokine receptor expression. To address this, we selected all tumor entities for which [^68^Ga]Pentixafor-PET images of at least 4 patients were available to us and compared PET-derived SLR. We detected minor differences in mean expression levels but could not define tumor entities with especially high levels of spleen [^68^Ga]Pentixafor uptake compared to others (Fig. 2A). However, we observed a great variance of spleen [^68^Ga]Pentixafor uptake within each tumor type, identifying patients within entities with manifold higher uptake compared to other patients with the same disease (Fig. 2A). Further, we hypothesized that systemic treatment could potentially influence receptor expression. Hence, we compared prior systemically treated patients to those who had not undergone treatment before imaging. We did not see any changes of spleen [^68^Ga]Pentixafor uptake in association with systemic treatment (Fig. 2B).

### Spleen [^68^Ga]Pentixafor uptake is not associated with clinical outcomes

**Fig. 3:**
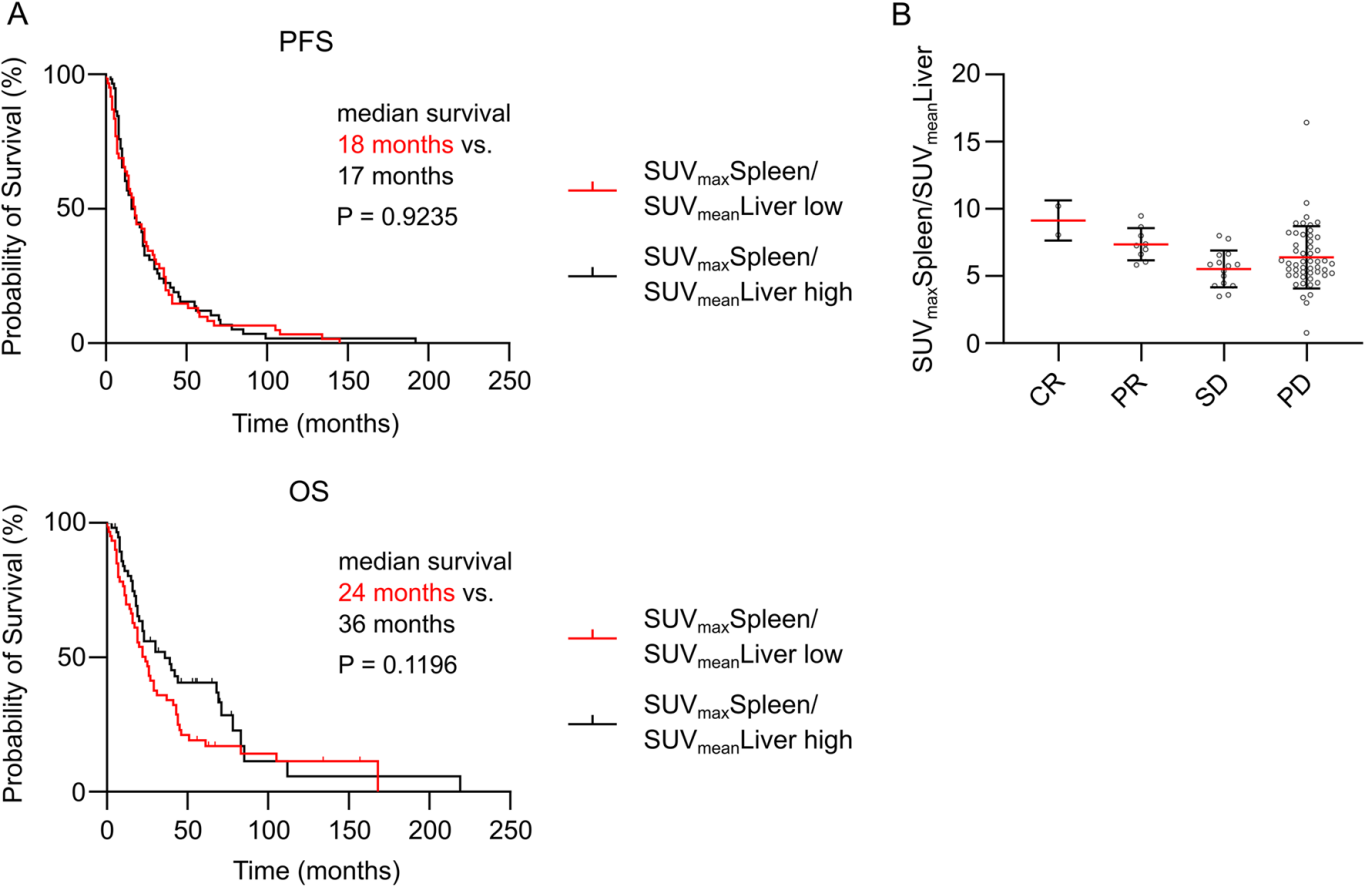
Spleen [^68^Ga]Pentixafor uptake does not correlate with clinical outcomes in solid tumor patients. **A** Kaplan–Meier survival curves of progression-free survival (PFS) and overall survival (OS) of all solid tumor patients in the study separated by the median into two groups of low and high spleen-to-liver ratios (SUV_max_Spleen/SUV_mean_Liver), PFS: low, *n* = 61, high, *n* = 58; OS: low, *n* = 61, high, *n* = 54. **B** Comparison of spleen-to-liver ratios (SUV_max_Spleen/SUV_mean_Liver) with remission status of all patients, complete remission (CR), *n* = 2; partial remission (PR), *n* = 9; stable disease (SD), *n* = 15; progressive disease (PD), *n* = 51. All statistical analyses were performed using log rank (Mantel–Cox) test (**A**) and one-way ANOVA with Tukey’s correction for multiple comparisons (**B**). *P* values as indicated on the graphs

From the observed wide variance of spleen CXCR4 expression levels, the question emerged, whether these differences were associated with patients’ clinical outcomes. Thus, we compared progression-free survival (PFS) and overall survival (OS) by separating all patients according to the median SLR for [^68^Ga]Pentixafor into two groups of low and high spleen CXCR4 expression. When comparing these groups, we saw similar probabilities of survival in both groups (Fig. 3A). Due to our approach of including all solid cancer patients, we faced a rather heterogenic composition of a variety of entities (Table 1). To rule out effects of differently proportioned allocation of entities to the two groups (low vs. high spleen [^68^Ga]Pentixafor uptake) we separated patients within each tumor entity the same way, according to the median, whenever there were enough evaluable patients to compare at least 3 versus 3 patients in each expression group. Hence, we assessed survival in adrenal cancer, DSRCT, neuroendocrine tumor, NSCLC, pancreatic adenocarcinoma and SCLC patients. We could not observe significant differences in PFS/OS in aforementioned entities when comparing low vs. high spleen [^68^Ga]Pentixafor uptake (Additional file 1: Figure S1). Additionally, we compared patients in the upper quartile of spleen CXCR4 expression to the lower quartile of all patients and, wherever possible, within entities and similarly observed no differences in PFS/OS (Additional file 1: Figure S2). We furthermore assessed whether disease remission was associated with splenic [^68^Ga]Pentixafor uptake. Hence, we compared the remission status of all patients and grouped patients into complete remission, partial remission, stable disease and progressive disease. Similar to the survival data, no differences in remission status could be seen in relation to spleen CXCR4 expression as measured by spleen [^68^Ga]Pentixafor uptake.

### Elevated spleen [^68^Ga]Pentixafor uptake is positively associated with leukocyte and/or platelet counts in neuroendocrine tumors, NSCLC and SCLC

**Fig. 4:**
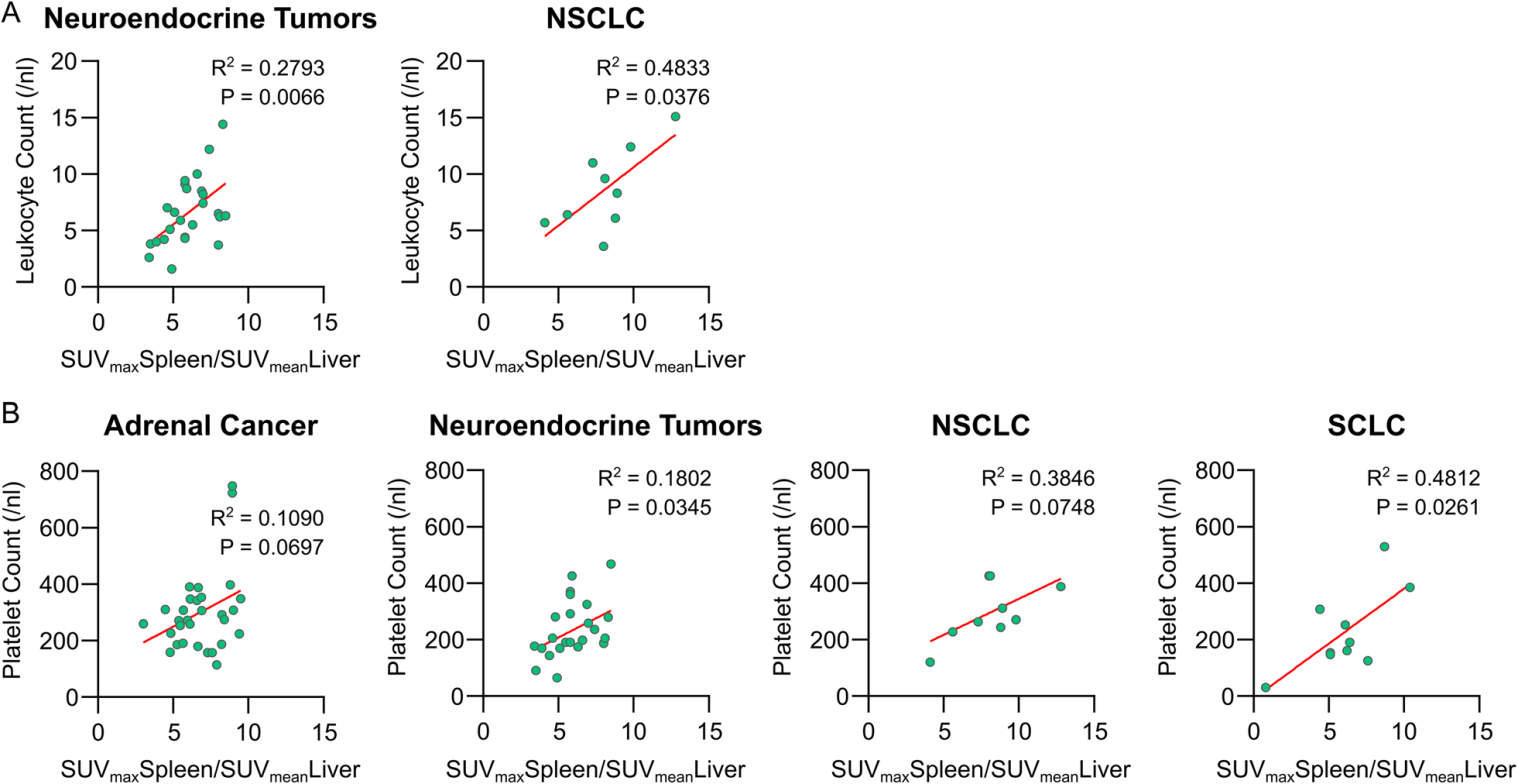
Spleen [^68^Ga]Pentixafor uptake is positively associated with peripheral blood leukocyte and/or platelet counts in neuroendocrine tumors, NSCLC and SCLC. **A** Correlation of spleen-to-liver ratio (SUV_max_Spleen/SUV_mean_Spleen) with peripheral leucocyte count in patients with neuroendocrine tumors, *n* = 25 and NSCLC, *n* = 9. **B** Correlation of spleen-to-liver ratio (SUV_max_Spleen/SUV_mean_Spleen) with peripheral platelet count in patients with adrenal tumors, *n* = 31; neuroendocrine tumors, *n* = 25; NSCLC, *n* = 9 and SCLC, *n* = 13. All statistical analyses were performed using simple linear regression. *R*^2^ and *P* values as indicated on the graphs

We hypothesized differences in spleen CXCR4 expression measured by [^68^Ga]Pentixafor-PET could potentially be associated with tumor metabolism/activity. Hence, we evaluated laboratory parameters of all patients from the day of imaging and correlated the respective SLR with serum C-reactive protein (CRP) (mg/dl), serum lactate-dehydrogenase (LDH) (U/l), hemoglobin levels (g/dl), peripheral leukocyte count (/nl) and peripheral platelet count (/nl). When investigating serum CRP, serum LDH and hemoglobin levels, we could not detect any significant association with spleen [^68^Ga]Pentixafor uptake (Additional file 1: Figures S3–S6). However, in patients with neuroendocrine tumors and NSCLC we could observe a positive correlation of splenic [^68^Ga]Pentixafor uptake with peripheral blood leukocyte counts (neuroendocrine tumors: *p* = 0.0066; NSCLC: *p* = 0.0376; Fig. 4A). Furthermore, platelet counts correlated with receptor expression in neuroendocrine tumors and SCLC (neuroendocrine tumors: *p* = 0.0363; SCLC: *p* = 0.0261) and there was a trend of an association with platelet counts in adrenal cancer and NSCLC (adrenal cancer: *p* = 0.0697; NSCLC: *p* = 0.0748; Fig. 4B and Additional file 1: Figure S7). Platelet counts were moreover associated with spleen [^68^Ga]Pentixafor uptake independently of the tumor entity when analyzing the entire patient cohort (*p* = 0.0006; Additional file 1: Figure S3).

## Discussion

There is some evidence implicating elevated serum CRP [32], elevated serum LDH [33, 34] and anemia [35] as markers for clinical outcomes in solid tumors. Furthermore, elevated leukocyte counts have been linked to unfavorable clinical outcomes in solid tumors [36–39] and thrombocytosis is associated with adverse outcomes in many cancers [40–42]. As for most patients the aforementioned parameters were available, we investigated the hypothesis that elevated [^68^Ga]Pentixafor uptake of spleen could be associated with an activated state of spleen and/or immune activity and clinical outcome. In our cohort, we could not discover significant correlations of serum CRP, serum LDH or blood hemoglobin levels with spleen [^68^Ga]Pentixafor uptake. However, platelet counts were positively associated with splenic drug uptake, regardless of tumor entity. When investigating single entities, in patients with neuroendocrine tumors, NSCLC and SCLC platelet and/or leukocyte counts were positively associated with splenic uptake. In both entities imaging of CXCR4 using [^68^Ga]Pentixafor was previously demonstrated to be feasible [21, 43, 44]. Furthermore, platelet counts showed a strong tendency of association in adrenal cancer and SCLC patients, too. Many data suggest tumors directly or indirectly stimulate thrombopoiesis through production of thrombopoietin (TPO) or interleukins [40, 45]. Interleukin 6 (IL-6) is among those interleukins most commonly elevated in the tumor microenvironment and has been shown to stimulate thrombopoiesis [46]. Furthermore, in different cell types, regulation of the release of IL-6 by the CXCR4/SDF-1 axis was demonstrated [47, 48]. CXCR4 and IL-6 are key-regulators of inflammation and leucocyte migration in immunity and cancer [49, 50] and could potentially play a role in systemic immunity. Thus, spleen [^68^Ga]Pentixafor uptake could possibly be a marker for spleen CXCR4 expression and tumors with a higher inflammatory component and tumors that rely more heavily on a functional tumor microenvironment that recruits platelets and leukocytes. These complex regulatory mechanisms of the tumor microenvironment could also help to interpret lacking differences in serum CRP, serum LDH and hemoglobin levels and explain why differences were not seen in all entities as the mechanisms involved might not be as relevant across different cancers or specifically in each patient’s tumors.

Additionally, we found no differences in survival outcomes when investigating drug uptake to the spleen, indicating that splenic [^68^Ga]Pentixafor uptake is not a single suitable parameter to predict patients’ outcomes. However, more entities could potentially show associations of splenic drug uptake with laboratory parameters or clinical outcome, as for many entities too few patients were available to us to make a solid conclusion.

Taken together, tumor-associated differences in spleen [^68^Ga]Pentixafor uptake might be more relevant in some entities rather than all solid cancers. Moreover, based on our data, we think that spleen [^68^Ga]Pentixafor uptake should be interpreted on a patient-to-patient basis even within the same tumor entity. However, more data needs to be collected to assess whether spleen [^68^Ga]Pentixafor uptake is related to clinical outcomes and to estimate the clinical and therapeutic consequences, especially in patients undergoing immunotherapy.

Of course, due to the retrospective nature of this study, further limitations need to be taken into consideration. We did not have the chance of follow-up PET/CT imaging of our patients and can thus not compare, whether changes in spleen CXCR4 expression during/after treatment would implicate changes in clinical parameters or outcomes. In addition, patients were partially selected for imaging with [^68^Ga]Pentixafor due to their relapsed disease and unfavorable prognosis. This could have led to a selection bias. Moreover, for some entities only very few patients were available and a bias due to these small cohorts needs to be taken into account. Lastly, other factors influencing spleen uptake values in patients, e.g. technical reasons during the application, different pharmacokinetics and possibly differences in hepatic metabolism and uptake consequently influencing the SLR need to be taken into account. However, our study provides the first investigation of spleen [^68^Ga]Pentixafor uptake in a large cohort of 145 solid tumor patients and 5 non-cancer control patients. We show spleen [^68^Ga]Pentixafor uptake is not associated with survival, but positively associated with leukocyte and/or platelet counts in neuroendocrine, NSCLC and SCLC, possibly attributed to an activated state of spleen and/or elevated tumor metabolism/activity.

## Conclusion

Spleen [^68^Ga]Pentixafor uptake was not associated with PFS, OS or current disease remission status in a large cohort of 145 solid cancer patients. Furthermore, prior systemic treatment was not associated with increased or decreased spleen [^68^Ga]Pentixafor uptake levels. However, we identified platelet counts and/or leukocyte counts to be positively correlated with spleen [^68^Ga]Pentixafor uptake in neuroendocrine tumors, NSCLC and SCLC. Further investigations need to clarify the mechanism underlying the highly variable [^68^Ga]Pentixafor uptake within the spleen of cancer patients.

## Methods

### Patient inclusion criteria

We retrospectively evaluated all [^68^Ga]Pentixafor-PET images of solid cancer patients, available to us. In total, we could collect data of 145 patients with solid cancers representing 27 different tumor entities. Furthermore, [^68^Ga]Pentixafor-PET images of 5 control patients were available to us. Of these, 3 patients were diagnosed with Conn’s adenoma and 2 patients were diagnosed as healthy. The patient characteristics of this cohort are detailed in Table 1.

### Synthesis of [^68^Ga]Pentixafor and imaging using [^68^Ga]Pentixafor

All patients in this study were imaged between January of 2013 and October of 2018 in the facilities of Universitätsklinikum Würzburg, Würzburg, Germany and Klinikum rechts des Isar, Munich, Germany. We retrospectively evaluated [^68^Ga]Pentixafor-PET images from our database of patients that had been previously published. Synthesis and imaging of patients was performed as previously described [15, 21, 43, 51–53]. All patients were imaged 60 min after an injection and dosage ranged from of 64 to 345 MBq (available from 141 patients and 5 controls). Patients received a dosage of MBq of Pentixafor. [^68^Ga]Pentixafor was administered in accordance to the German Medicinal Products Act, AMG §13 2b, and with permission of the responsible regulatory authorities (Regierung von Oberbayern, Regierung von Oberfranken and Regierung von Unterfranken) and the respective ethics committees of Universitätsklinikum Würzburg and Klinikum rechts des Isar.

### Calculation of spleen-to-liver ratio (SLR)

Splenic [^68^Ga]Pentixafor uptake was measured by placing a volume of interest (VOI) around the whole spleen and applying isocontouring until no extra-splenic structures were included in the VOI. Liver uptake was measured by placing a VOI of at least 30 mm in healthy hepatic tissue. Spleen and liver VOI were matched. To account for interindividual variance of tracer biodistribution, we normalized spleen uptake by calculating a spleen-to-liver ratio (SLR). SLR was derived by dividing the maximum SUV of the spleen (SUV_max_Spleen) by the mean SUV of the liver (SUV_mean_Liver).

### Formation of groups for survival analysis

To assess the probability of survival, we conducted two separate analyses. First, we calculated the median SLR for [^68^Ga]Pentixafor as explained above using GraphPad Prism Version 9.0.1 (GraphPad Prism Software, La Jolla, CA, USA).within the respective cohorts. Patients were then allocated according to the median to a high and low group of spleen CXCR4 expression. Secondly, we assessed larger cohorts by forming two groups representing the highest and lowest quartile of spleen CXCR4 expression as measured by SLR for [^68^Ga]Pentixafor.

### Laboratory parameters

We collected laboratory parameters from the day of imaging, wherever these were available to us, for serum C-reactive protein (mg/dl) (*n* = 132), serum lactate-dehydrogenase (LDH) (U/l) (*n* = 126), hemoglobin levels (g/dl) (*n* = 143), peripheral leukocyte count (/nl) (*n* = 143) and peripheral platelet count (/nl) (*n* = 143).

### Disease stage and remission status

To determine disease stage we collected information on the TNM stage of all solid cancer patients and grouped patients into limited disease stage, whenever they were classified with T1 or T2 stage and did not have any distant metastases (M0). Accordingly, patients with T3 or T4 stage, as well as subjects with distant metastases (M1) were classified as advanced stage. All patients for whom the exact allocation to limited or advanced disease was not possible were classified as unknown. We additionally evaluated patients upon their subsequent patient records and grouped them wherever possible into complete remission, partial remission, stable disease and progressive disease.

### Systemic treatment prior to imaging of patients

We retrospectively evaluated patient records. Prior treatments included chemotherapy, radiotherapy and surgery, as well as combinations of the aforementioned. One patient additionally received immunotherapy. To evaluate whether systemic treatment would influence [^68^Ga]Pentixafor uptake, we compared those patients without ongoing systemic chemotherapy to those who had undergone systemic chemotherapy within the last month prior to imaging.

### Statistics and graphical display of data

All analyses were performed using GraphPad Prism Version 9.0.1. (GraphPad Prism Software, La Jolla, CA, USA) and figures were constructed using InkScape Version 0.92.3 (Inkscape Project, open source). Quantitative variables were described as mean values ± 1 standard deviation. PFS and OS were estimated using the Kaplan–Meier method and compared between groups with the log-rank test. Associations between continuous variables were analyzed with a linear regression model and depicted as scatter plots including regression line and R2 value, indicating the coefficient of determination. For all analysis, two-sided *p* values < 0.05 were considered significant.

## Supplementary Information

**Additional file 1**. Supplemental Figures 1–7, which provide additional Kaplan-Meier survival curves and correlations of laboratory parameters as mentioned in the results section.

## Data Availability

The clinical data contains potentially personal information and is stored with the authors. The datasets used and/or analyzed during the current study are available from the corresponding author upon reasonable request.

## Abbreviations

CR: Complete remission
CRP: C-reactive protein
CT: Computed tomography
CXCR4: C-X-C motif chemokine receptor type 4
DSRCT: Desmoplastic small round cell tumor
IL-6: Interleukin 6
LDH: Lactate dehydrogenase
MDSC: Myeloid-derived suppressor cells
NSCLC: Non-small cell lung cancer
OS: Overall survival
PD: Progressive disease
PET: Positron emission tomography
PFS: Progression-free survival
PR: Partial remission
SCLC: Small cell lung cancer
SD: Stable disease
SDF-1: Stromal cell-derived factor 1
SLR: Spleen-to-liver ratio
SUV: Standardized uptake value
TPO: Thrombopoietin
VOI: Volume of interest
