# Supplementary material for: Investigation of spleen CXCR4 expression by [68Ga]Pentixafor PET in a cohort of 145 solid cancer patients
Source: EJNMMI Res. 2021 Aug 21;11:77. doi: 10.1186/s13550-021-00822-6 (PMC8380222; doi:10.1186/s13550-021-00822-6)
Supplement: Supplementary file 1 — Additional file 1. Supplemental Figures 1–7, which provide additional Kaplan-Meier survival curves and correlations of laboratory parameters as mentioned in the results section. [file 13550_2021_822_MOESM1_ESM.pdf]

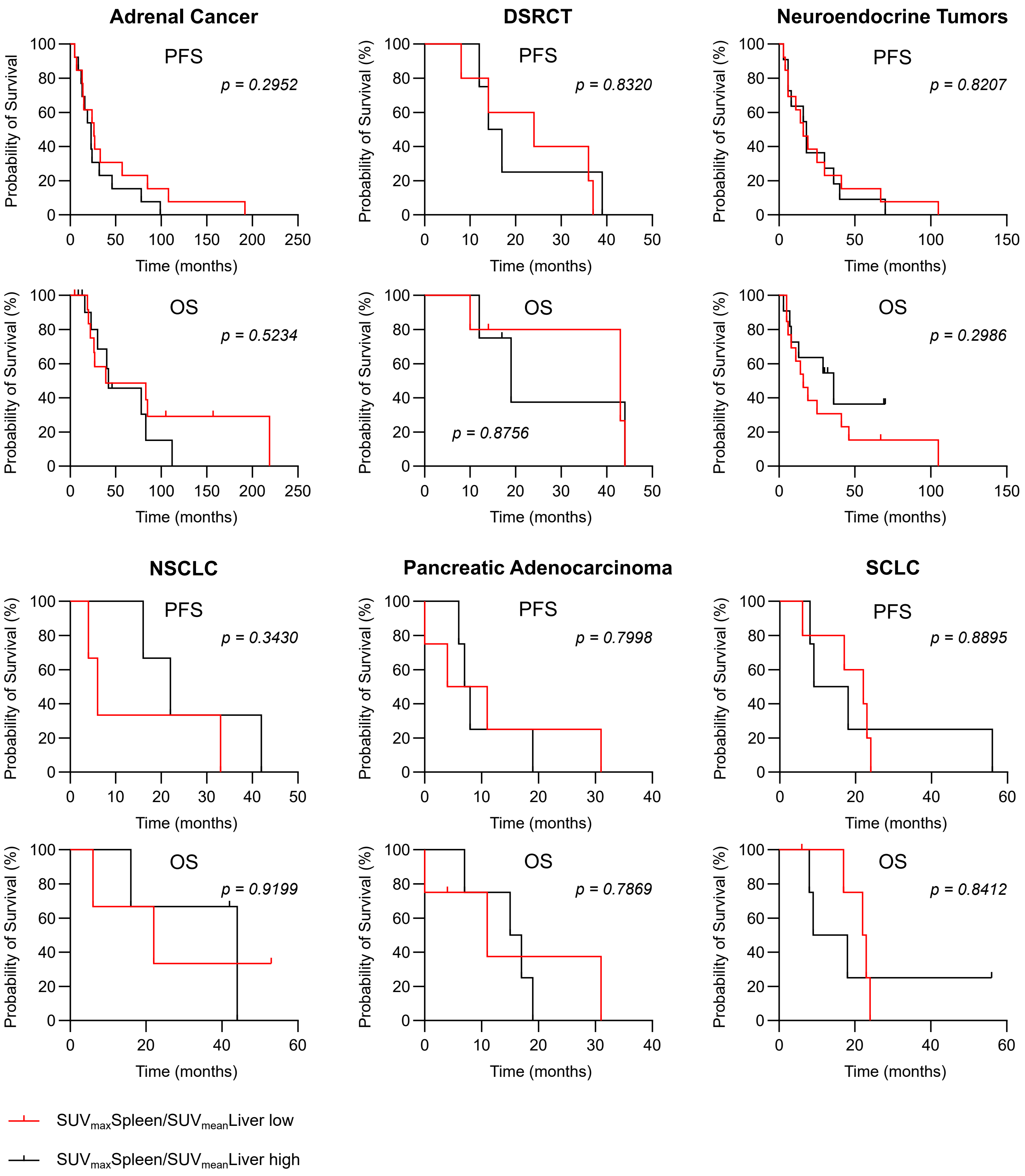

Supplemental Figure 1. Spleen $[^{68}Ga]$Pentixafor uptake is not associated with PFS/OS in different entities. Kaplan-Meier survival curves of progression-free survival (PFS) and overall survival of different solid tumor entities, comparing patients with low to patients with high spleen-to-liver ratio (SUV_{max}Spleen/SUV_{mean}Liver), adrenal cancer, PFS: low, n = 13, high, n = 13, OS: low, n = 13, high, n = 13; DSRCT, PFS: low, n = 5, high, n = 4, OS: low, n = 5, high, n = 4; neuroendocrine tumors, PFS: low, n = 13, high, n = 11, OS: low, n = 13, high, n =11; NSCLC, PFS: low, n = 3, high, n = 3, OS: low, n = 3, high, n = 3; pancreatic adenocarcinoma, PFS: low, n = 4, high, n = 4, OS: low, n = 4, high, n = 4; SCLC, PFS: low, n = 5, high, n = 4, OS: low, n = 5, n = 4.
All statistical analyses were performed using log-rank (Mantel-Cox) test. P values as indicated on the graphs.

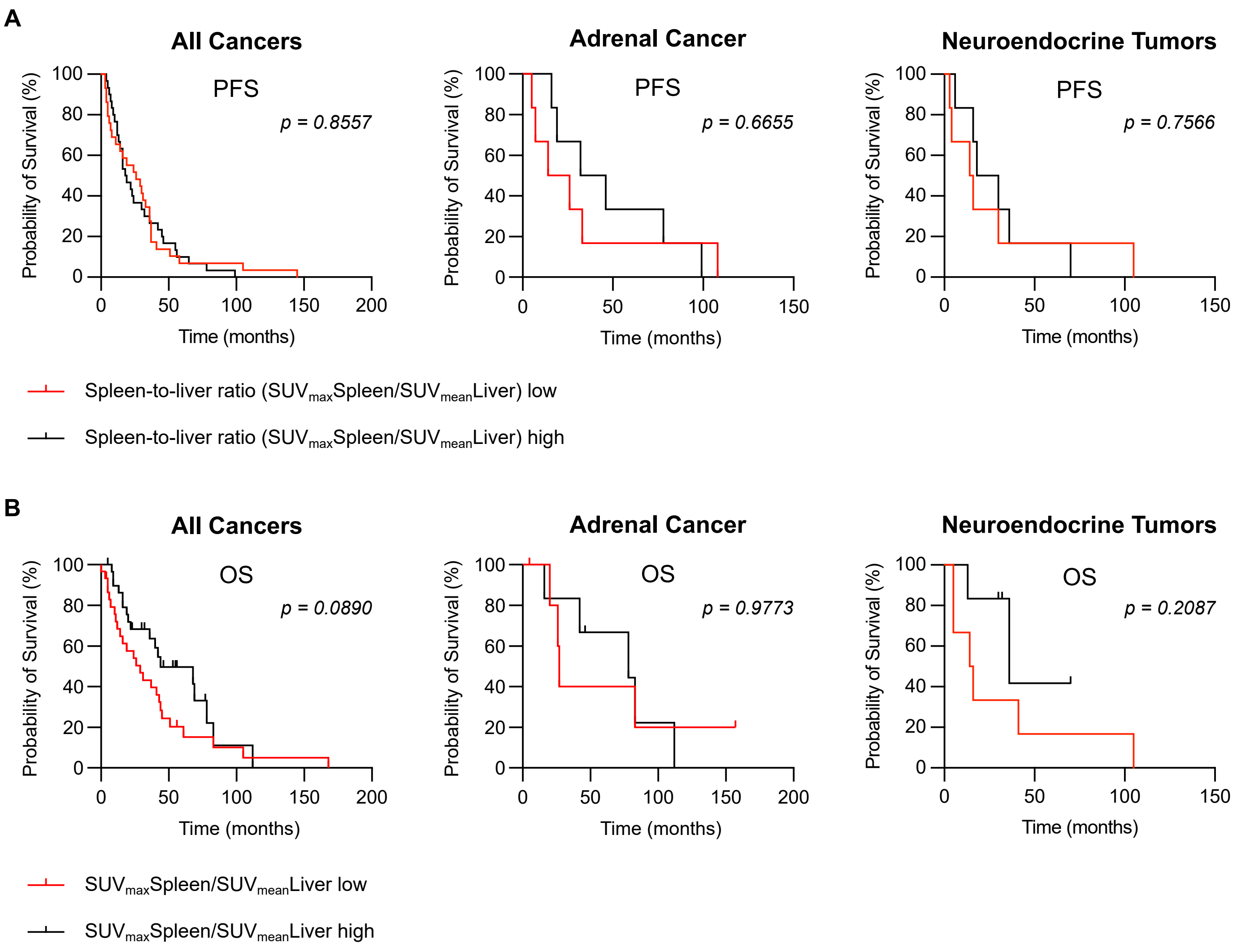

Supplemental Figure 2. Spleen [^{68}Ga]Pentixafor uptake is not associated with PFS/OS in all entities, or adrenal cancers and neuroendocrine tumors, when comparing the highest to the lowest quartile of spleen CXCR4 expression.

A Kaplan-Meier survival curves of progression-free survival, comparing the quartile with highest to the quartile of lowest spleen-to-liver ratio (SUV_{max}Spleen/SUV_{mean}Liver). All cancers: low, n = 30, high, n = 30; adrenal cancers: low, n = 6, high, n = 6; neuroendocrine tumors: low, n = 6, high, n = 6.

B Kaplan-Meier survival curves of overall survival, comparing the quartile with highest to the quartile of lowest spleen-to-liver ratio (SUV_{max}Spleen/SUV_{mean}Liver). All cancers: low, n = 30, high, n = 30; adrenal cancers: low, n = 6, high, n = 6; neuroendocrine tumors: low, n = 6, high, n = 6.

All statistical analyses were performed using log-rank (Mantel-Cox) test. P values as indicated on the graphs.

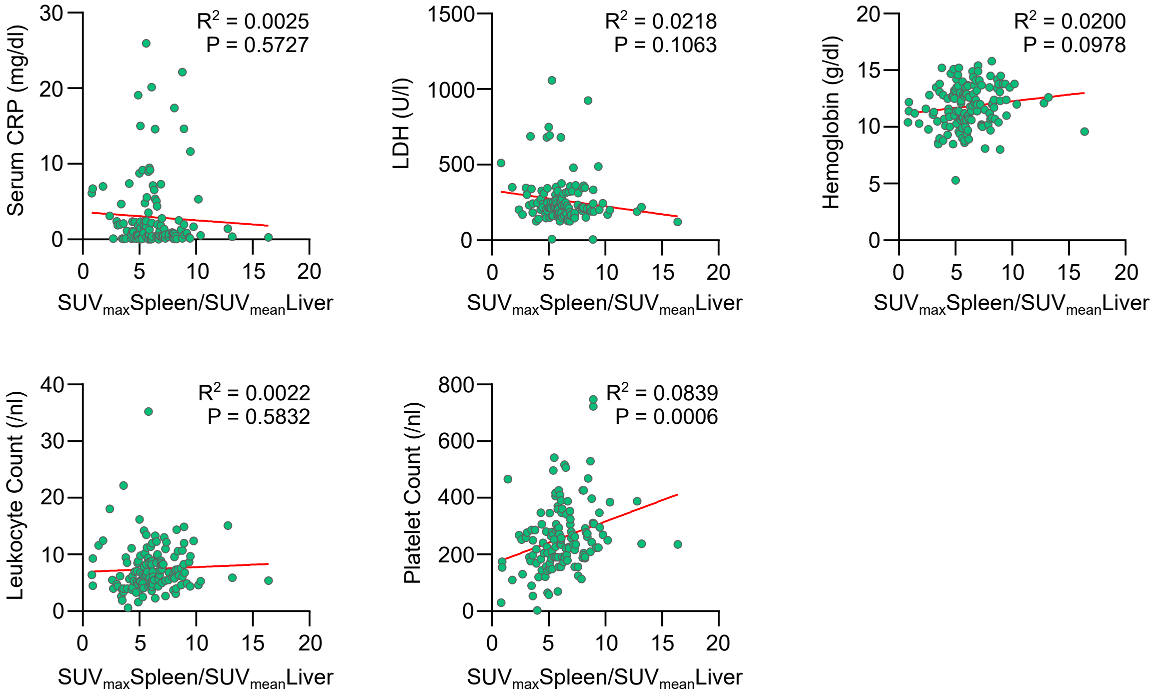

Supplemental Figure 3. Spleen [68Ga]Pentixafor is positively associated with platelet counts in solid cancers.

Correlations of spleen-to-liver ratios (SUV_{max}Spleen/SUV_{mean}Liver) of all patients in this study with serum CRP levels (mg/dl), n = 128; serum LDH levels (U/l), n = 121; blood hemoglobin levels (g/dl), n = 138; peripheral leucocyte count (/nl), n = 138 and peripheral platelet count (/nl), n = 138.

All statistical analyses were performed using simple linear regression. R^2 and P values as indicated on the graphs.

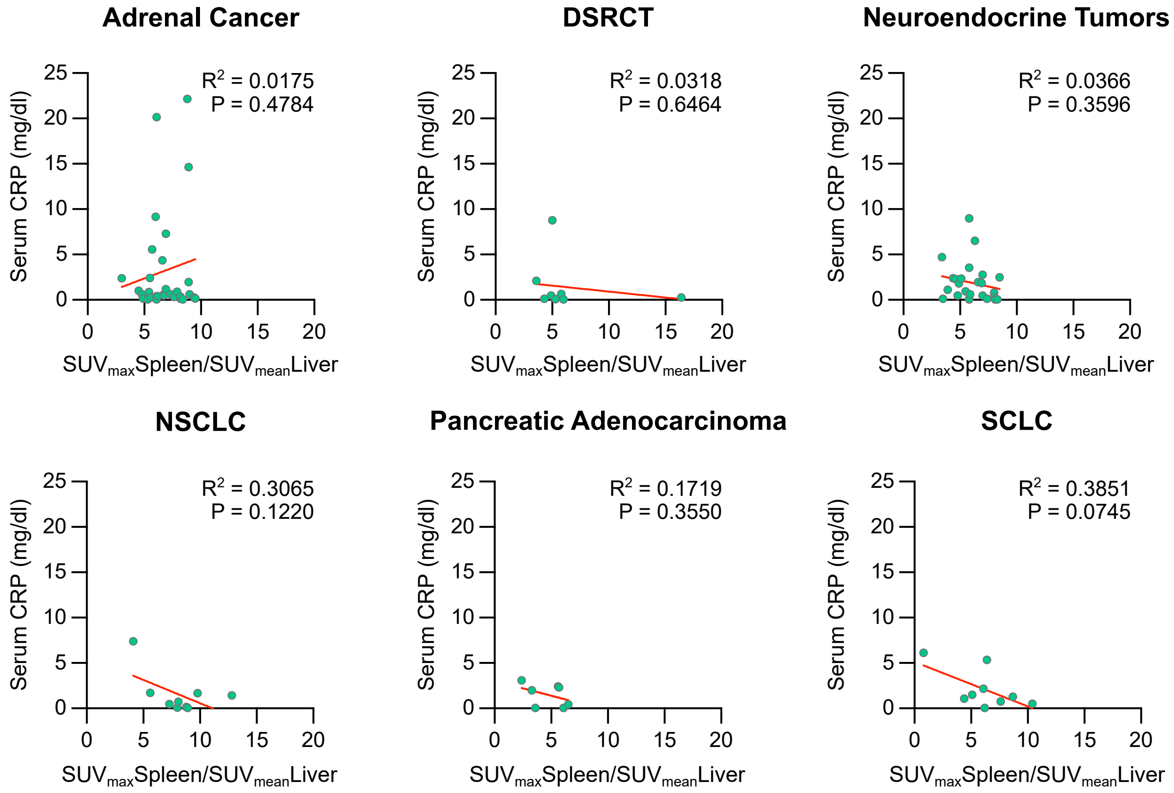

Supplemental Figure 4. Spleen [^{68}Ga]Pentixafor uptake is not associated with serum CRP levels.
Correlations of spleen-to-liver ratios (SUV_{max}Spleen/SUV_{mean}Liver) with serum CRP levels (mg/dl) in patients with adrenal cancer, n = 31; DSRCT, n = 9; neuroendocrine tumor, n = 25; NSCLC, n = 9; pancreatic adenocarcinoma, n = 7 and SCLC, n = 9.
All statistical analyses were performed using simple linear regression. R^2 and P values as indicated on the graphs.

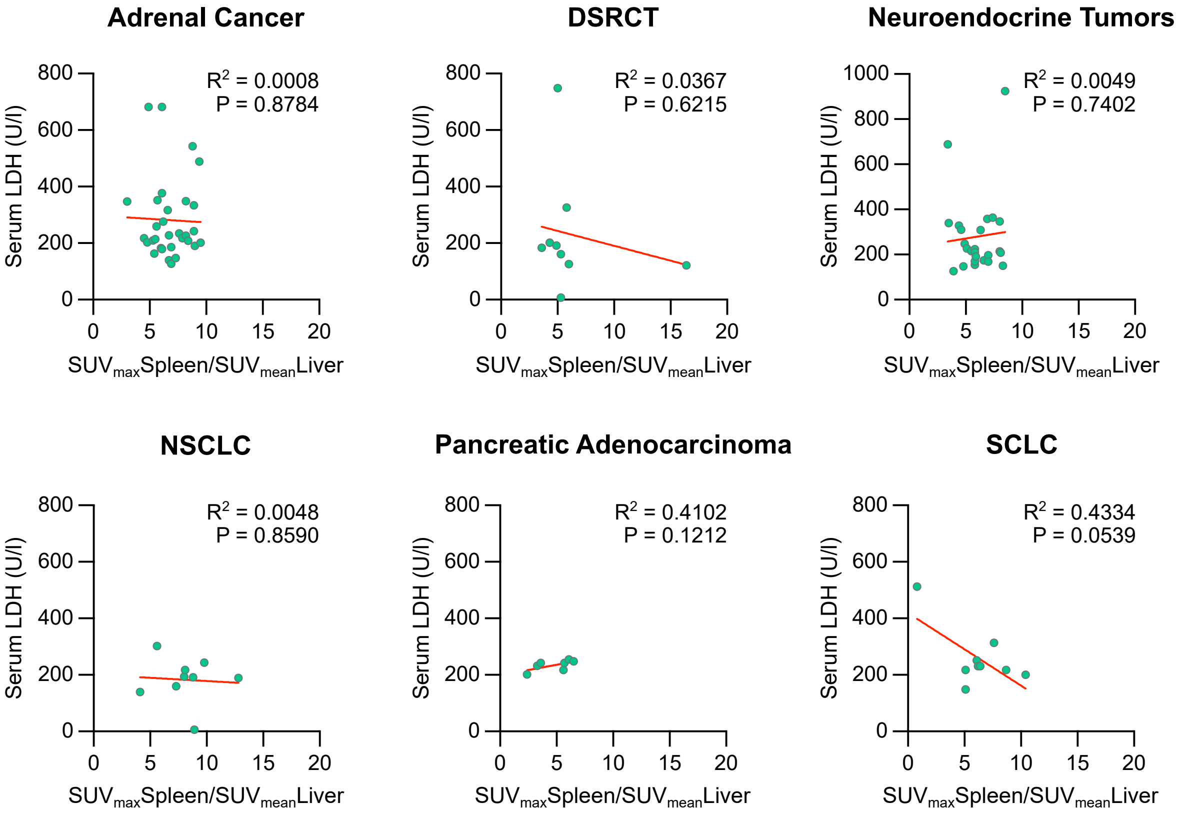

Supplemental Figure 5. Spleen [68Ga]Pentixafor uptake is not associated with serum LDH levels. Correlations of spleen-to-liver ratios (SUV_{max}Spleen/SUV_{mean}Liver) with serum LDH levels (U/l) in patients with adrenal cancer, n = 31; DSRCT, n = 9; neuroendocrine tumor, n = 25; NSCLC, n = 9; pancreatic adenocarcinoma, n = 7 and SCLC, n = 9.

All statistical analyses were performed using simple linear regression. R^2 and P values as indicated on the graphs.

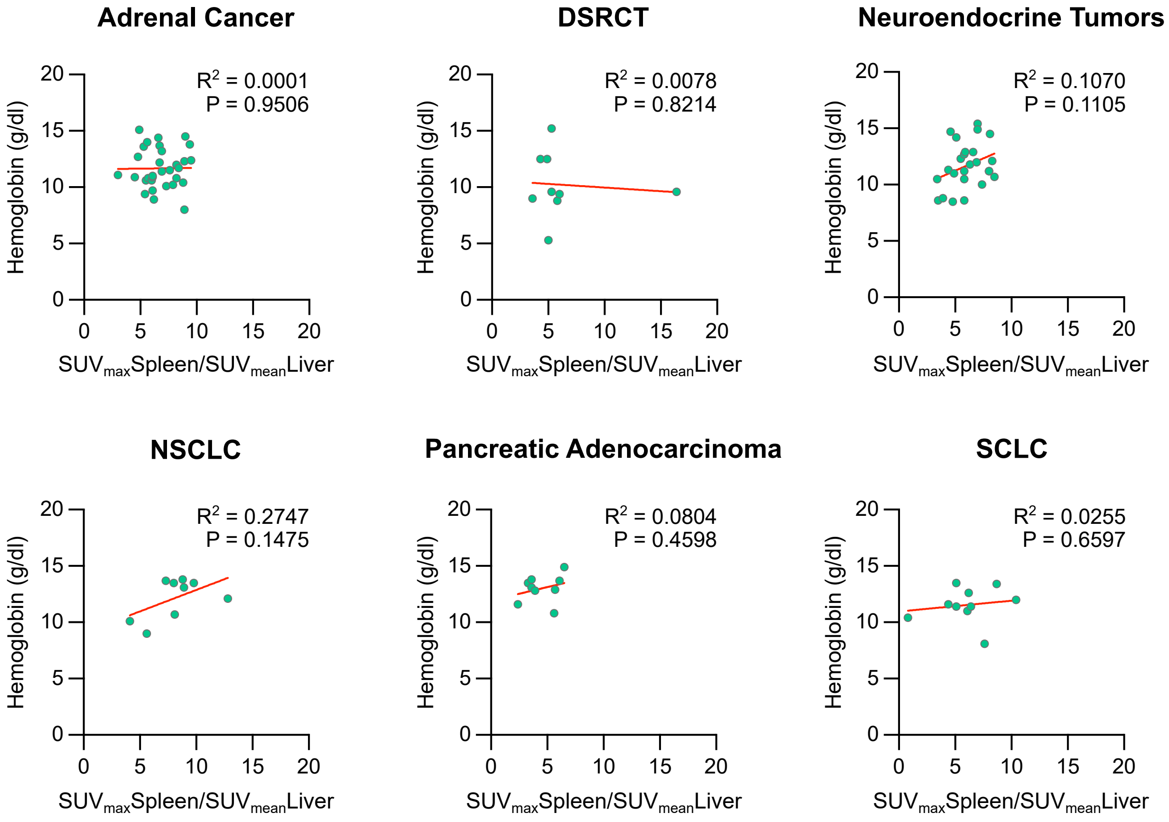

Supplemental Figure 6. Spleen [^{68}Ga]Pentixafor uptake is not associated with blood hemoglobin levels. Correlations of spleen-to-liver ratios (SUV_{max}Spleen/SUV_{mean}Liver) with blood hemoglobin levels (g/dl) in patients with adrenal cancer, n = 31; DSRCT, n = 9; neuroendocrine tumors, n = 25; NSCLC, n = 9; pancreatic adenocarcinoma, n = 7 and SCLC, n = 10.

All statistical analyses were performed using simple linear regression. R^2 and P values as indicated on the graphs.

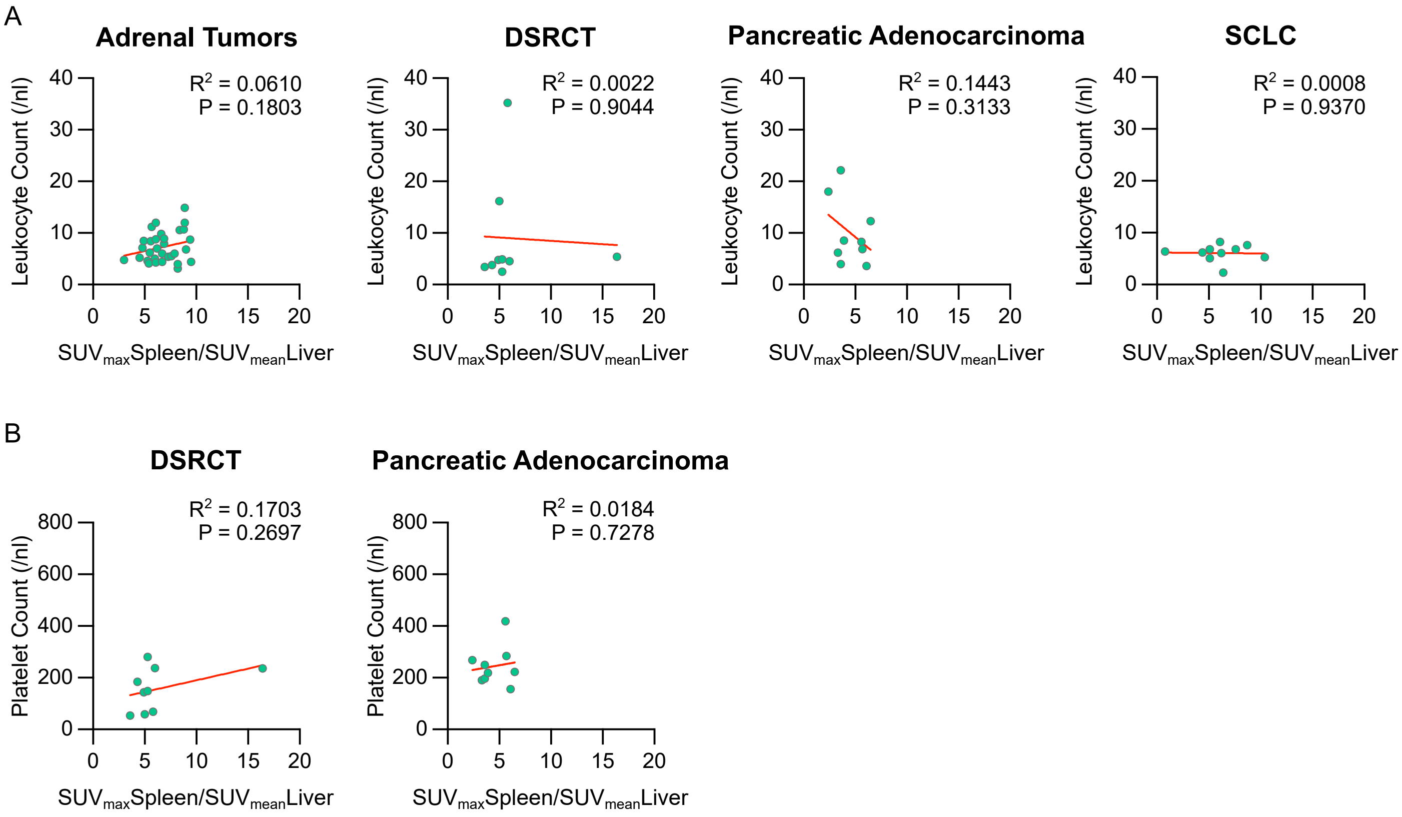

Supplemental Figure 7. Spleen [^{68}Ga]Pentixafor is not associated with leucocyte count and/or platelet counts in some entities.

A Correlations of spleen-to-liver ratios (SUV_{max}Spleen/SUV_{mean}Spleen) with peripheral leucocyte count in patients with adrenal cancer, n = 31; DSRCT, n = 9; pancreatic adenocarcinoma, n = 9 and SCLC, n = 10.

B Correlation of spleen-to-liver ratios (SUV_{max}Spleen/SUV_{mean}Spleen) with peripheral platelet count in patients with DSRCT, n = 9 and pancreatic adenocarcinoma, n = 9.

All statistical analyses were performed using simple linear regression. R^2 and P values as indicated on the graphs.
